# Endoscopic management of long-standing rectovaginal fistula caused by foreign bodies

**DOI:** 10.1055/a-2344-8608

**Published:** 2024-07-03

**Authors:** Li Wang, Zu-Qiang Liu, Pei-Rong Xu, Lu Yao, Hao Hu, Quan-Lin Li, Ping-Hong Zhou

**Affiliations:** 1 92323 Endoscopy Center and Endoscopy Research Institute, Zhongshan Hospital, Fudan University, Shanghai, China; 2 Endoscopy Center, Shanghai Geriatric Medical Center, Shanghai, China; 3 Shanghai Collaborative Innovation Center of Endoscopy, Shanghai, China

A 32-year-old woman was admitted, presenting with a 30-year history of rectovaginal fistula (RVF). At the age of 1 year, the patient inadvertently ingested a paperclip, resulting in fecal discharge from the vagina, and no intervention was implemented.

Colonoscopy revealed a 0.8-cm fistula above the dentate line on the anterior rectal wall ( Fig. 1Fig. 1 **a** ). Transanal endoscopic closure was proposed after a multidisciplinary evaluation ( Video 1Video 1 ).

**Fig. 1:**
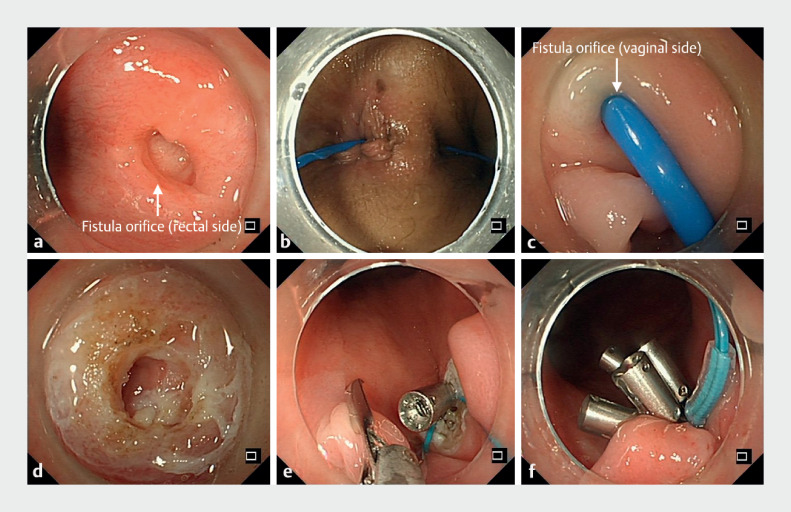
**Fig. 1** Endoscopic management of rectovaginal fistula. **a** Colonoscopy revealed a 0.8-cm fistula (arrowhead) above the dentate line on the anterior rectal wall. **b, c** A bougie inserted through the rectal fistula opening was exposed within the vagina. **d** The mucosal epithelium and scar tissue within the fistula orifice and sinus tract were destroyed with argon ion coagulation. **e, f** After confirming no bleeding, purse-string suturing was used to close the rectal fistula.

A long medical history of rectovaginal fistula caused by swallowing a foreign body was successfully treated with endoscopic purse-string suturing.Video 1Video 1

Upon bimanual examination, a cord-like structure extending from the anterior wall of the rectum to the posterior wall of the vagina was palpable. A bougie inserted through the rectal fistula opening was exposed within the vagina ( Fig. 1Fig. 1 **b, c** ). Argon ion coagulation was used to destroy the mucosal epithelium and scar tissue within the fistula orifice and sinus tract ( Fig. 1Fig. 1 **d** ). After confirming no bleeding, purse-string suturing was used to close the rectal fistula ( Fig. 1Fig. 1 **e, f** ). The procedure duration was 30 minutes.

The patient recovered uneventfully and was discharged on postoperative day 2. The patient did not experience any discomfort during the 2-year postoperative follow-up.

The main causes of RVF are obstetric trauma, chronic inflammatory bowel disease, pelvic floor or rectal surgery, trauma, or radiation therapy, and swallowed foreign bodies are a rare cause 11 . Although various medical and surgical (including transanal, transvaginal, and transperineal) methods have been used to manage RVFs, the treatment remains a challenge due to its tendency to recur. Innovative transanal endoscopic surgery has been proposed for the treatment of RVF, but endoscopic purse-string suturing has not previously been reported for the treatment of RVF 22 . Here, we present the first report of a case of a long medical history of RVF caused by swallowing a foreign body, which was successfully treated with endoscopic purse-string suturing. This report provides an innovative, minimally invasive treatment approach for patients with RVF.

Endoscopy_UCTN_Code_TTT_1AQ_2AB

## Notes

Endoscopy E-Videos https://eref.thieme.de/e-videos
*E-Videos* is an open access online section of the journal *Endoscopy* , reporting on interesting cases and new techniques in gastroenterological endoscopy. All papers include a high-quality video and are published with a Creative Commons CC-BY license. Endoscopy E-Videos qualify for HINARI discounts and waivers and eligibility is automatically checked during the submission process. We grant 100% waivers to articles whose corresponding authors are based in Group A countries and 50% waivers to those who are based in Group B countries as classified by Research4Life (see: https://www.research4life.org/access/eligibility/ ). This section has its own submission website at https://mc.manuscriptcentral.com/e-videos .
